# Incorporating Present-on-Admission Indicators in Medicare Claims to Inform Hospital Quality Measure Risk Adjustment Models

**DOI:** 10.1001/jamanetworkopen.2021.8512

**Published:** 2021-05-12

**Authors:** Elizabeth W. Triche, Xin Xin, Sydnie Stackland, Danielle Purvis, Alexandra Harris, Huihui Yu, Jacqueline N. Grady, Shu-Xia Li, Susannah M. Bernheim, Harlan M. Krumholz, James Poyer, Karen Dorsey

**Affiliations:** 1 Center for Outcomes Research and Evaluation, Yale New Haven Hospital, New Haven, Connecticut; 2 Section of Cardiovascular Medicine, Department of Internal Medicine, Yale School of Medicine, New Haven, Connecticut; 3 Section of General Medicine, Department of Internal Medicine, Yale School of Medicine, New Haven, Connecticut; 4 Department of Health Policy and Administration, Yale School of Public Health, New Haven, Connecticut; 5 Centers for Medicare & Medicaid Services (CMS), Woodlawn, Maryland; 6 Section of General Pediatrics, Department of Internal Medicine, Yale School of Medicine, New Haven, Connecticut

## Abstract

**Importance:**

Present-on-admission (POA) indicators in administrative claims data allow researchers to distinguish between preexisting conditions and those acquired during a hospital stay. The impact of adding POA information to claims-based measures of hospital quality has not yet been investigated to better understand patient underlying risk factors in the *International Statistical Classification of Diseases and Related Health Problems, Tenth Revision* setting.

**Objective:**

To assess POA indicator use on Medicare claims and to assess the hospital- and patient-level outcomes associated with incorporating POA indicators in identifying risk factors for publicly reported outcome measures used by the Centers for Medicare & Medicaid Services (CMS).

**Design, Setting, and Participants:**

This comparative effectiveness study used national CMS claims data between July 1, 2015, and June 30, 2018. Six hospital quality measures assessing readmission and mortality outcomes were modified to include POA indicators in risk adjustment models. The models using POA were then compared with models using the existing complications-of-care algorithm to evaluate changes in risk model performance. Patient claims data were included for all Medicare fee-for-service and Veterans Administration beneficiaries aged 65 years or older with inpatient hospitalizations for acute myocardial infarction, heart failure, or pneumonia within the measurement period. Data were analyzed between September 2019 and March 2020.

**Main Outcomes and Measures:**

Changes in patient-level (C statistics) and hospital-level (quintile shifts in risk-standardized outcome rates) model performance after including POA indicators in risk adjustment.

**Results:**

Data from a total of 6 027 988 index admissions were included for analysis, ranging from 491 366 admissions (269 209 [54.8%] men; mean [SD] age, 78.2 [8.3] years) for the acute myocardial infarction mortality outcome measure to 1 395 870 admissions (677 158 [48.5%] men; mean [SD] age, 80.3 [8.7] years) for the pneumonia readmission measure. Use of POA indicators was associated with improvements in risk adjustment model performance, particularly for mortality measures (eg, the C statistic increased from 0.728 [95% CI, 0.726-0.730] to 0.774 [95% CI, 0.773-0.776] when incorporating POA indicators into the acute myocardial infarction mortality measure).

**Conclusions and Relevance:**

The findings of this quality improvement study suggest that leveraging POA indicators in the risk adjustment methodology for hospital quality outcome measures may help to more fully capture patients’ risk factors and improve overall model performance. Incorporating POA indicators does not require extra effort on the part of hospitals and would be easy to implement in publicly reported quality outcome measures.

## Key Points

**Question:**

Could present-on-admission indicators enhance risk models used by the Centers for Medicare & Medicaid Services to assess acute myocardial infarction, heart failure, and pneumonia mortality and readmission measures?

**Findings:**

In this comparative effectiveness study including all Medicare fee-for-service beneficiaries hospitalized for acute myocardial infarction, heart failure, or pneumonia at acute care hospitals, the incorporation of present-on-admission indicators into patient-level and hospital-level 30-day mortality and readmission risk models was associated with modest improvement of discrimination in model performance.

**Meaning:**

These results suggest that accounting for preexisting conditions in hospital quality outcome data could enhance mortality and readmission measure risk models while incurring no additional burden to health care professionals.

## Teaser

This comparative effectiveness study uses national Medicare data on hospitalizations to evaluate whether the inclusion of present-on-admission indicators is associated with improved discrimination in Centers for Medicare & Medicaid Services (CMS) hospital quality risk models for 30-day mortality and readmission.

## Introduction

Many of the outcome measures used by the Centers for Medicare & Medicaid Services (CMS) use Medicare fee-for-service (FFS) administrative claims to determine patient case mix. These measures generate most of their risk variables using claims diagnosis codes for the index hospitalization and for encounters in the 12 months prior to the index hospitalization. Risk adjustment should account for differences in patients’ conditions before or at the time of admission, but not for conditions that develop during the hospitalization and could be attributed to the care received. Conditions coded on claims within the 12 months before the index admission are always considered present before the time of admission; however, for conditions coded only on the index admission claim, it can be difficult to distinguish whether certain comorbidities are complications of care arising from a hospital stay or are preexisting conditions present on admission. Therefore, many current CMS measures use an algorithm for excluding diagnoses coded only during the index admission from the risk model if they could be potential complications of care.

The introduction of POA indicators in administrative claims presents an opportunity to more accurately distinguish conditions present at the time of admission from complications of care. In 2007, CMS mandated that all Inpatient Prospective Payment System hospitals other than critical access hospitals (CAHs) and Maryland hospitals use present-on-admission (POA) indicators for each diagnosis code on a claim to signify whether a patient had the condition at the time of admission.^1^ This mandate was expanded to include Maryland hospitals in 2014.^2^ This change in reporting enhances the information that can be used in risk models; by helping to distinguish risk variables that were present upon index admission from complications of care that occurred during the admission, POA indicators may more accurately discern preexisting conditions from potential signals of poor quality.^3,4,5^

Literature on the use of POA indicators is limited. Researchers have primarily investigated the use of POA indicators in the *International Classification of Diseases, Ninth Revision (ICD-9)* coding system. Most of these studies referenced data collected prior to the CMS mandate for reporting POA indicators on claims, leading to significant variability in reporting across hospitals. Furthermore, most of the existing literature has examined the use of POA indicators within a health system or at a state-level; large-scale national assessment of risk model performance using POA indicators has yet to be conducted using *International Statistical Classification of Diseases and Related Health Problems, Tenth Revision (ICD-10)* codes.

Given that the use of POA indicators in risk models has the potential to improve the accuracy of risk adjustment, the aims of this study were to assess: (1) POA indicator use on Medicare claims overall and among non-CAHs and CAHs separately; and (2) the hospital- and patient-level outcomes associated with incorporating POA indicators in 6 of CMS’s publicly reported outcome measures (acute myocardial infarction [AMI] readmission, AMI mortality, heart failure [HF] readmission, HF mortality, pneumonia [PN] readmission, and PN mortality).

## Methods

### Data Sources

In this comparative effectiveness study, data included claims for all Medicare FFS and Veterans Administration (VA) beneficiaries aged 65 years or older with inpatient hospitalizations for AMI, HF, or PN between July 1, 2015, and June 30, 2018. VA beneficiaries were eligible for inclusion in these measures regardless of Medicare FFS enrollment or whether they were hospitalized in a VA hospital or non-VA short-term acute care hospital. The cohorts were defined by index admission claims (*ICD-10 Clinical Modification* [*ICD-10-CM*] codes) with a principal discharge diagnosis for each respective condition: AMI for the AMI readmission and AMI mortality measures, HF for the HF readmission and mortality measures, or PN for the PN readmission and mortality measures, consistent with CMS’s publicly reported hospital inpatient measures.^6^ For eligible beneficiaries, inpatient (Part A) and outpatient (Part B) claims for the 12 months prior to admission were used to identify risk variables (eg, comorbid conditions). To be eligible, beneficiaries must have been: (1) enrolled in Medicare FFS Parts A and B for the 12 months prior to the admission and Part A during the index admission, or have been VA beneficiaries, to ensure a full year of risk adjustment data; and (2) enrolled in Medicare FFS or with the VA for the 30-day postdischarge period or until death, to capture the outcome. Patients who left care facilities against medical advice were excluded from the mortality and readmission measure analyses. Patients who were transferred from another acute care hospital were excluded from the mortality measure analyses. In readmission measure analyses, patients who died during admission or who were transferred to another acute care hospital were excluded.^7,8^

The Yale University human investigation committee reviewed the study protocol and exempted it from informed consent requirements because the research involved no more than minimal risk and could not be practically carried out otherwise. This study followed the International Society for Pharmacoeconomics and Outcomes Research (ISPOR) reporting guideline.

### Study Outcome Measures

The measures included in this study are AMI readmission, AMI mortality, HF readmission, HF mortality, PN readmission, and PN mortality. The 3 readmission measures each assess a binary outcome of all-cause unplanned readmissions to any acute care hospital within 30 days of discharge from the index hospitalization. The CMS planned readmission algorithm version 4.0 was used to exclude any planned readmissions based on procedures or diagnoses that are usually scheduled.^8^ The 3 mortality measures each assess a binary outcome of all-cause mortality within 30 days of the first day of the index admission.^8^

### Risk Adjustment Variables and POA Indicators

CMS’s publicly reported readmission and mortality measures risk-adjust for patient comorbidities using diagnosis and procedure codes from index admission claims and historical claims in the 12 months prior to the index admission. The diagnosis codes are primarily grouped based on CMS hierarchical condition categories,^9,10^ which include 201 unique condition categories. For current measure specifications, diagnoses and corresponding condition categories coded during the index admission are used as risk variables only if they are not considered potential complications of care (CoC)^8^ as defined by a clinically vetted algorithm. Using these criteria, each of the measures has a unique set of comorbidity risk variables that a patient may have on an index admission claim, up to 27 possible comorbidities for AMI mortality, 24 for HF mortality, 36 for PN mortality, 31 for AMI readmission, 37 for HF readmission, and 41 for PN readmission.

For these analyses, we used all current measure specifications with the addition of POA status for each risk variable. In claims where POA status was not reported, such as on certain claims from CAHs, we retained CMS’s existing CoC algorithm for risk adjustment; here, conditions that were only coded during the index admission and could be potential CoC were excluded from risk adjustment. Note that our POA methods did not affect model risk factors identified within the 12 months prior to admission. For full details about measure risk models, refer to the reports posted on Hospital Compare.^11^

### Statistical Analysis

To evaluate the outcomes associated with incorporating POA indicators on the 6 CMS measures of interest, we first assessed how consistently hospitals coded POA on index admission claims in accordance with CMS’s mandate. We included all risk variables derived from diagnosis POA = “Y” indicator or on an “always POA” list, which is a subset of codes on CMS’s POA-exempt list^1^ that clinicians have determined always reflect health status at admission (such as subsequent encounter or sequela codes). See eAppendix 1 in the Supplement for further details. We examined the variability in the usage of POA across hospitals within each of the measures. Given that CAHs are not required to report POA, we compared the proportions of claims with missing POA indicators separately for CAHs and non-CAHs. We considered a claim to be missing POA indicators if POA status was not reported for any diagnosis code on the claim.

Next, we examined the change in the mean number of comorbidity risk variables identified per beneficiary on a given index admission claim after incorporating POA. For instance, for the AMI mortality model that includes 27 potential risk variables, we aimed to determine if the mean number of comorbidity risk variables identified for a given beneficiary on an index admission claim would increase when incorporating POA indicators, compared with the existing model using only the CoC algorithm. We then compared model performance (C statistics) at the patient level for logistic regression models with and without POA. We additionally present calibration plots (ie, observed outcome rate deciles plotted against the mean estimated probability deciles). A wider range of estimated probabilities suggests a better predictive ability and relatively better model performance.

To assess the outcomes associated with incorporating POA codes at the hospital level, we assessed quintile shifts in hospital-level risk-standardized mortality rates and risk-standardized readmission rates for the current CMS models that use the CoC algorithm, compared with models using POA indicators. Only hospitals with at least 25 claims were included, as hospitals with fewer admissions are not reported on Hospital Compare by CMS.

Risk-standardized mortality and readmission rates were calculated through hierarchical logistic regression models.^7^ Briefly, these hierarchical models account for clustering within and between hospitals. Models are adjusted for sex, age, selected comorbidities, and a hospital-specific random effects intercept. The risk-standardized mortality or readmission rate is calculated as the ratio of the number of projected outcomes to the number of expected outcomes (ie, death or readmission) multiplied by the national unadjusted rate of the given outcome.^7^

Data were analyzed between September 2019 and March 2020. All analyses were conducted using SAS statistical software version 9.4 (SAS Institute).

## Results

### Cohort Descriptions

Each measure’s cohort size, the number of hospitals included in the analyses, and patient demographic information are summarized in Table 1. Results show a large volume of index admissions across the measures, ranging from 491 366 to 1 395 870 index admissions in the AMI mortality (269 209 [54.8%] men; mean [SD] age, 78.2 [8.3] years) and PN readmission (677 158 [48.5%] men; mean [SD] age, 80.3 [8.7] years) measures, respectively. Moreover, all measures included more than 4100 hospitals nationwide.

**Table 1.** Cohort Description for Acute Myocardial Infarction, Heart Failure, and Pneumonia Mortality and Readmission Measures

Characteristic	Admissions, No. (%)					
	Mortality			Readmission		
	Acute myocardial infarction (n = 491 366)	Heart failure (n = 1 055 330)	Pneumonia (n = 1 330 877)	Acute myocardial infarction (n = 502 198)	Heart failure (n = 1 252 347)	Pneumonia (n = 1 395 870)
Hospitals, No.	4304	4661	4723	4150	4665	4727
With ≥25 claims	2387	3690	4254	2197	3770	4254
Male	269 209 (54.8)	506 550 (48.0)	604 523 (45.4)	276 668 (55.1)	603 089 (48.2)	677 158 (48.5)
Female	222 157 (45.2)	548 780 (52.0)	726 354 (54.6)	225 530 (44.9)	649 258 (51.8)	718 712 (51.5)
Age, mean (SD), y	78.2(8.3)	80.8 (8.5)	80.5 (8.7)	77.9 (8.3)	80.6 (8.5)	80.3 (8.7)

### Variability in Hospital Use of POA Indicators

Results describing hospital usage of POA indicators are shown in Table 2. More than 99% of all non-CAH facilities across all 6 measure cohorts had fewer than 20% of their claims with POA indicators missing for all diagnoses (eg, AMI readmission for non-CAH: <20% POA indicators missing, 3172 [99.9%] claims). CAHs were less consistent in the use of POA indicators (AMI readmission for CAH: <20% POA indicators missing, 798 [81.8%] claims). However, among CAHs, more than 80% of the AMI mortality and readmission measure cohorts and 78% of the remaining measure cohorts consistently used POA (ie, <20% of their claims missing POA).

**Table 2.** Summary of Hospital-Level Present-on-Admission (POA) Indicator Use by Critical Access Hospital Status for AMI, HF, and PN Readmission and Mortality Measures, July 2015 to June 2018

Measure cohort	POA indicator data available	Admissions, No. (%)		
		Non-CAH	CAH	All hospitals
**Readmission**				
AMI	Hospitals, No.	3174	976	4150
	<20% missing	3172 (99.9)	798 (81.8)	3970 (95.7)
	≥20 to <80% missing	2 (0.1)	42 (4.3)	44 (1.1)
	≥80% missing	0	136 (13.9)	136 (3.3)
HF	No.	3327	1338	4665
	<20% missing	3324 (99.9)	1048 (78.3)	4372 (93.7)
	≥20 to <80% missing	3 (0.1)	116 (8.7)	119 (2.6)
	≥80% missing	0	174 (13)	174 (3.7)
PN	No.	3373	1354	4727
	<20% missing	3369 (99.9)	1064 (78.6)	4433 (93.8)
	≥20 to <80% missing	4 (0.1)	121 (8.9)	125 (2.6)
	≥80% missing	0	169 (12.5)	169 (3.6)
**Mortality**				
AMI	No.	3220	1084	4304
	<20% missing	3217 (99.9)	874 (80.6)	4091 (95.1)
	≥20 to <80% missing	3 (0.1)	61 (5.6)	64 (1.5)
	≥80% missing	0	149 (13.7)	149 (3.5)
HF	No.	3325	1336	4661
	<20% missing	3324 (100)	1047 (78.4)	4371 (93.8)
	≥20 to <80% missing	1 (0)	119 (8.9)	120 (2.6)
	≥80% missing	0	170 (12.7)	170 (3.6)
PN	No.	3370	1353	4723
	<20% missing	3366 (99.9)	1064 (78.6)	4430 (93.8)
	≥20 to <80% missing	4 (0.1)	117 (8.6)	121 (2.6)
	≥80% missing	0	172 (12.7)	172 (3.6)

Abbreviations: AMI, acute myocardial infarction; CAH, critical access hospital; HF, heart failure; POA, present on admission; PN, pneumonia.

### Patient-Level Model Outcomes

Table 3 provides information on the incremental outcomes associated with POA indicators on patient-level models. With the incorporation of POA indicators, the mean (SD) number of comorbidities identified per beneficiary during an index admission increased from 7.3 (4.0) to 8.0 (3.9), from 11.4 (4.7) to 12.2 (4.3), and from 10.7 (5.4) to 11.9 (SD 5.0) for the AMI, HF, and PN readmission measures, respectively. Similarly, for the AMI, HF, and PN mortality measures, the mean (SD) number of comorbidities identified per beneficiary during an index admission increased from 6.5 (3.2) to 7.1 (3.2), from 7.9 (3.2) to 8.4 (2.9), and from 8.1 (4.2) to 9.3 (4.0), respectively (Table 3). Furthermore, C statistics improved for both AMI readmission (current model, 0.658; 95% CI, 0.656-0.660 vs model with POA, 0.662; 95% CI 0.660-0.664) and AMI mortality (current model, 0.728; 95% CI, 0.726-0.730 vs model with POA, 0.774; 95% CI, 0.773-0.776), for HF mortality (current model, 0.684; 95% CI, 0.683-0.686 vs model with POA, 0.694; 95% CI, 0.692-0.695), and for PN mortality (current model, 0.720; 95% CI, 0.719-0.721 vs model with POA, 0.743; 95% CI, 0.742-0.744). The C statistics were nearly identical for HF readmission and PN readmission between models with and without POA indicators. No measures showed a decrease in the number of comorbidities included in risk adjustment, and no risk model C statistics decreased after incorporating POA.

**Table 3.** Performance of Patient-Level AMI, HF, and PN Readmission and Mortality Measure Models Using Only the CoC Algorithm and Using POA Indicators^a^

Measure	Patients, No.	Current model using the CoC algorithm^b^		Model with POA^c^	
		Comorbidities per admission, mean (SD)	C statistic (95% CI)	Comorbidities per admission, mean (SD)	C statistic (95% CI)
**Readmission**					
AMI	502 198	7.3 (4.01)	0.658 (0.658-0.660)	8.0 (3.9)	0.662 (0.660-0.664)
HF	1 252 347	11.4 (4.7)	0.611 (0.610-0.612)	12.2 (4.3)	0.611 (0.609-0.612)
PN	1 395 870	10.7 (5.4)	0.637 (0.635-0.638)	11.9 (5.0)	0.638 (0.636-0.639)
**Mortality**					
AMI	491 366	6.5 (3.2)	0.728 (0.726-0.730)	7.11 (3.2)	0.774 (0.773-0.776)
HF	1 055 330	7.9 (3.2)	0.684 (0.683-0.686)	8.4 (2.9)	0.694 (0.692-0.695)
PN	1 330 877	8.1 (4.2)	0.720 (0.719-0.721)	9.3 (4.0)	0.743 (0.742-0.744)

Abbreviations: AMI, acute myocardial infarction; CAHs, critical access hospitals; CoC, complications of care; HF, heart failure; PN, pneumonia; POA, present on admission.

^a^ All models also adjusted for age, sex, and history of coronary artery bypass grafting or percutaneous coronary intervention; however, these variables are not included in the average number of comorbidities included in the model. Patient-level models were calculated using logistic regression.

^b^ Using current Centers for Medicare & Medicaid Services (CMS) algorithm to distinguish POA conditions from CoC.

^c^ Using POA indicator as primary method of distinguishing conditions present on admission from complications of care; if POA indicators missing, current CMS algorithm was used.

The calibration plots in the Figure suggest improved performance for the 3 mortality measures after incorporating POA compared with the current CMS models. There were no meaningful differences observed in the calibration plots across the 3 readmission measures when comparing models incorporating POA to models without POA.

**Figure.**
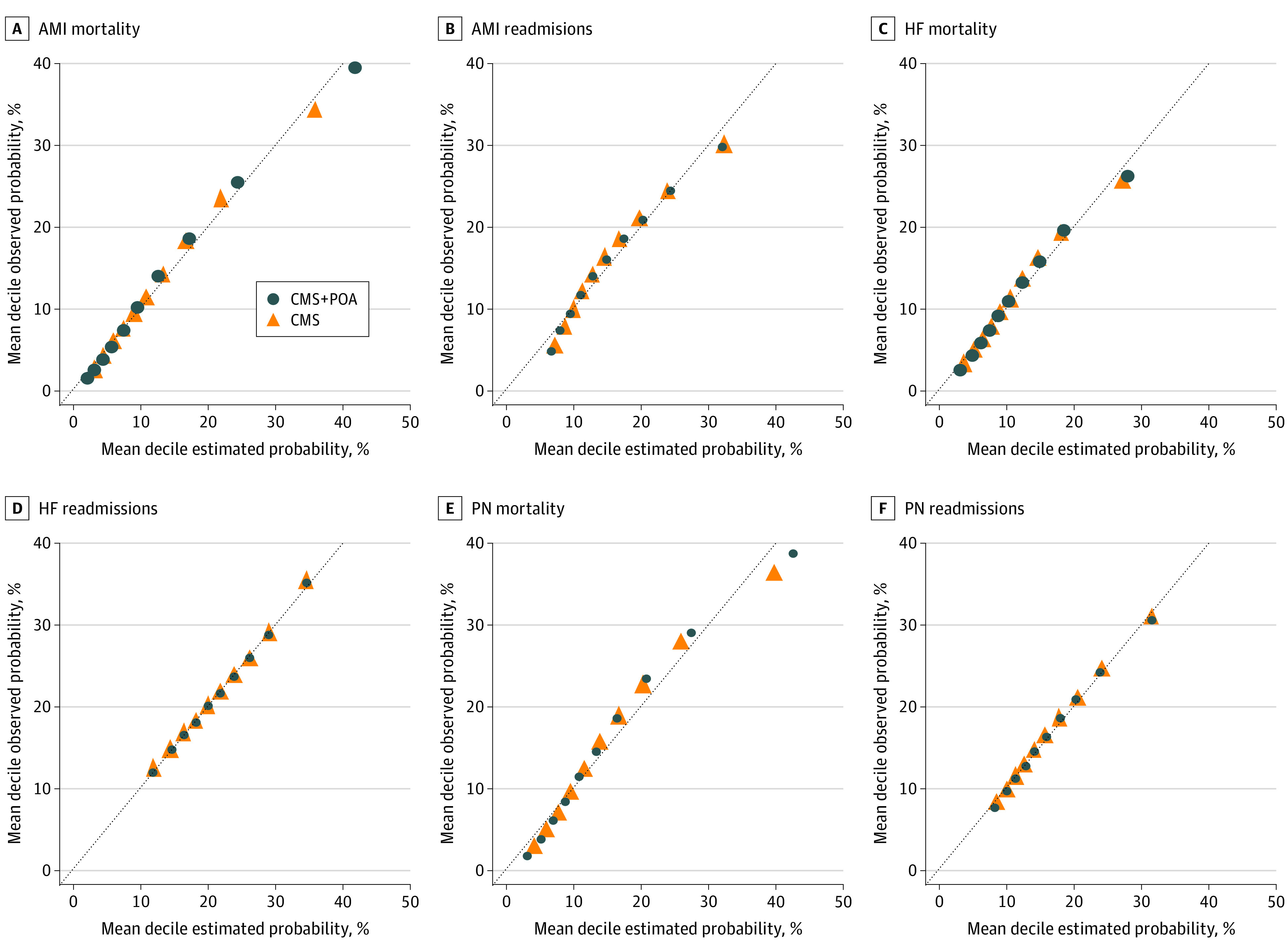
Calibration Plots Comparing 6 Hospital Quality Measures Used by Centers for Medicare & Medicaid Services (CMS) With Models Including Present-on-Admission (POA) Indicators AMI indicates acute myocardial infarction; HF, heart failure; PN, pneumonia.

### Hospital-Level Model Outcomes

Table 4 compares quintile shifts in hospital-level outcome rates (ie, the risk-standardized mortality and readmission rates) between current CMS models using only the CoC algorithm and models using POA indicators by CAH status. Detailed measure-specific tables are provided in eTables 1 through 6 in the Supplement. Across the 3 readmission measures, a large majority of hospitals remained in the same quintile of risk-standardized readmission rates when comparing models with and without POA indicators (eg, heart failure readmission: non-CAHs remaining in same quintile, 2850 [92.7%]; CAHs remaining in same quintile, 642 [92.5%]). None of the hospitals shifted more than 1 quintile. Mean differences in hospital-level risk-standardized readmission rates between models using the CoC algorithm vs POA were very small across all 3 readmission measures (eTables 1-3 in the Supplement). Across the 3 mortality measures, a considerable proportion of both non-CAHs and CAHs shifted 1 quintile. Only a small proportion of hospitals in the AMI and PN mortality measures shifted 2 quintiles; no hospitals shifted 2 quintiles in the HF mortality measure. None of the hospitals shifted more than 2 quintiles. Mean differences in hospital-level risk-standardized mortality rates between models with and without POA indicators were small across all 3 mortality measures, albeit larger than for the readmission measures (eTables 4-6 in the Supplement).

**Table 4.** Quintile Shifts in Risk-Standardized Outcome Rates With Addition of Present-on-Admission Indicators by CAH Status

Measure	Total Non-CAHs, No.	Non-CAHs, No. (% of total non-CAHs)		Total CAHs, No.	CAHs, No. (% of total CAHs)	
		Shifting 1 quintile	Shifting 2 quintiles		Shifting 1 quintile	Shifting 2 quintiles
**Readmission**						
AMI	2183	210 (9.6)	0	14	4 (28.6)	0
HF	3076	226 (7.3)	0	694	52 (7.5)	0
PN	3166	304 (9.6)	0	1088	126 (11.6)	0
**Mortality**						
AMI	2352	622 (26.4)	13 (0.6)	35	16 (45.8)	3 (8.6)
HF	3056	512 (16.8)	0	634	100 (15.8)	0
PN	3156	896 (28.4)	24 (0.8)	1089	294 (27.0)	7 (0.6)

Abbreviations: AMI, acute myocardial infarction; CAHs, critical access hospitals; HF, heart failure; PN, pneumonia.

## Discussion

In this study, we demonstrated that using POA indicators to distinguish between conditions present on admission and complications of care acquired during an index admission may expand the information about patients’ risk and modestly improve the statistical model used to profile hospital performance for publicly reported mortality and readmission measures. Model performance improvements were more substantial for the mortality measures compared with the readmission measures.

As expected, with the incorporation of POA indicators, we observed an increase in the number of comorbidity risk variables per beneficiary on an index admission. By incorporating POA indicators, many comorbidities that are currently excluded from risk adjustment in CMS outcome measures because they cannot be determined to be complications or comorbidities can be identified as present upon admission and therefore included in the risk models. This allows for improvement in model discrimination and greater face validity of the risk models. All patient-level models showed either no change or slight improvements in goodness of fit, suggesting that models that incorporate POA indicators at the index admission may better estimate the risk of readmission and mortality compared with current models that only use the CoC algorithm. There were minimal changes in hospital-level quintile shifts for the 3 readmission measures, but more variation in hospital-level performance was present across the 3 mortality measures, highlighting that mortality may be more influenced by patient-level clinical factors.

The findings of the current study demonstrate that hospitals have begun to consistently report POA indicators on Medicare FFS claims. We did not assess the validity of POA indicators; however, existing literature suggests that POA reporting is relatively consistent with medical record information.^12,13^ A 2011 study^14^ indicated that POA reporting was consistent for 74.3% of records when compared with masked medical record review in *ICD-9* data, and a 2009 study^12^ conducted in *ICD-10-AM* (Australian Modification) found a 93.4% agreement between POA coding and expert medical record review.

Other research has investigated the potential impact of POA on hospital rankings, as shifts in rankings could be a factor in hospital reimbursement if the outcome measures are included in CMS payment programs.^15^ Glance et al^5^ showed significant shifts in rankings with the addition of POA indicators to the Agency for Healthcare Research and Quality Inpatient Quality Indicator measures, though their findings were based on data collected several years before CMS’s POA reporting mandate. Consistent with our findings, which showed 27% of hospitals in the AMI mortality measure experiencing at least 1 quintile shift in performance, Goldman et al^15^ recently found that, in an AMI mortality cohort, approximately 25% of hospitals experienced a shift in rankings greater than 10%, though overreporting and underreporting of POA had little effect on hospital performance rankings. As noted previously, most of the studies reviewed were conducted at the health system or state level and focused on outcomes for specific diseases or procedures, limiting the generalizability of the findings.

The findings of this study are consistent with prior research on how POA indicators factor into risk models,^3,4,16^ although, to the best of our knowledge, this is the first study to show such evidence in risk models defined by *ICD-10* codes. One previous comparative effectiveness study on similar mortality cohorts^3^ showed improvements in risk models that included POA indicators in the *ICD-9* coding system. A subsequent study of select CMS payment measures similarly demonstrated an incremental benefit.^4^ Further research has suggested that, while including POA indicators may impact hospital quality rankings, these changes are not due to inaccuracies in POA reporting.^15,16^

The observed improvements in model performance for measures that are already in public reporting are promising. Incorporating POA indicators requires no additional reporting burden for hospitals and will likely also improve face validity of the measures by better capturing patients’ health status upon presentation for care. Furthermore, model improvements were consistent across the 6 outcome measures tested for this study, and analyses encompassed all Medicare FFS beneficiaries aged 65 years and older nationally.

### Limitations

Our study has several limitations. We did not assess the accuracy of POA coding by hospitals in this study. Previous research has suggested that the use of POA to account for hospital case mix may be prone to gaming if POA indicators are incorporated into measures that impact payment.^17,18^ There is some prior evidence that the accuracy of POA coding varies by diagnosis and type of hospital.^14^ However, audits of POA coding by CMS could help to discourage inaccurate coding and prevent gaming. Furthermore, a study by Goldman et al^15^ found that inaccuracies in POA coding did not account for changes in hospital rankings of AMI mortality based on models incorporating POA compared with models without POA.^15^

## Conclusions

Leveraging POA indicators for risk adjustment in hospital quality outcome measures may help to more fully capture patients’ risk factors and improve overall model performance. Incorporating POA indicators does not require extra effort on the part of hospitals and would be easy to implement in publicly reported quality outcome measures. The relatively small model enhancements seen with POA suggest that current measure methodology that does not use POA status is still a valid option for hospitals, such as CAHs, with lower POA reporting likely due to differential reporting requirements.

## Notes

Supplement. **eAppendix 1.** POA Exempt Codes **eAppendix 2.** Note on Quintile Shifts in eTables 1-6 **eTable 1.** Comparing the RSRRs from the Current CMS Model Without POA (Using Only the CoC Algorithm) to the CMS+POA Model Across Five Quintiles, AMI Readmission **eTable 2.** Comparing the RSRRs from the Current CMS Model Without POA (Using Only the CoC Algorithm) to the CMS+POA Model Across Five Quintiles, HF Readmission **eTable 3.** Comparing the RSRRs from the Current CMS Model Without POA (Using Only the CoC Algorithm) to the CMS+POA Model Across Five Quintiles, PN Readmission **eTable 4.** Comparing the RSMRs from the Current CMS Model Without POA (Using Only the CoC Algorithm) to the CMS+POA Model Across Five Quintiles, AMI Mortality **eTable 5.** Comparing the RSMRs from the Current CMS Model Without POA (Using Only the CoC Algorithm) to the CMS+POA Model Across Five Quintiles, HF Mortality **eTable 6.** Comparing the RSMRs from the Current CMS Model Without POA (Using Only the CoC Algorithm) to the CMS+POA Model Across Five Quintiles, PN Mortality **eTable 7.** Distributions of Differences in Risk-Standardized Rates With and Without POA for AMI, HF, and PN Readmission and Mortality Measures
