# Supplementary material for: Incorporating Present-on-Admission Indicators in Medicare Claims to Inform Hospital Quality Measure Risk Adjustment Models
Source: JAMA Netw Open. 2021 May 12;4(5):e218512. doi: 10.1001/jamanetworkopen.2021.8512 (PMC8116982; doi:10.1001/jamanetworkopen.2021.8512)
Supplement: Supplement. — eAppendix 1. POA Exempt Codes eAppendix 2. Note on Quintile Shifts in eTables 1-6 eTable 1. Comparing the RSRRs from the Current CMS Model Without POA (Using Only the CoC Algorithm) to the CMS+POA Model Across Five Quintiles, AMI Readmission eTable 2. Comparing the RSRRs from the Current CMS Model Without POA (Using Only the CoC Algorithm) to the CMS+POA Model Across Five Quintiles, HF Readmission eTable 3. Comparing the RSRRs from the Current CMS Model Without POA (Using Only the CoC Algorithm) to the CMS+POA Model Across Five Quintiles, PN Readmission eTable 4. Comparing the RSMRs from the Current CMS Model Without POA (Using Only the CoC Algorithm) to the CMS+POA Model Across Five Quintiles, AMI Mortality eTable 5. Comparing the RSMRs from the Current CMS Model Without POA (Using Only the CoC Algorithm) to the CMS+POA Model Across Five Quintiles, HF Mortality eTable 6. Comparing the RSMRs from the Current CMS Model Without POA (Using Only the CoC Algorithm) to the CMS+POA Model Across Five Quintiles, PN Mortality eTable 7. Distributions of Differences in Risk-Standardized Rates With and Without POA for AMI, HF, and PN Readmission and Mortality Measures [file jamanetwopen-e218512-s001.pdf]

Supplemental Online Content

Triche EW, Xin X, Stackland S, et al. Incorporating present-on-admission indicators in Medicare claims to inform hospital quality measure risk adjustment models. *JAMA Netw Open*. 2021;4(5):e218512. doi:10.1001/jamanetworkopen.2021.8512

eAppendix 1. POA Exempt Codes

eAppendix 2. Note on Quintile Shifts in eTables 1-6

eTable 1. Comparing the RSRRs from the Current CMS Model Without POA (Using Only the CoC Algorithm) to the CMS+POA Model Across Five Quintiles, AMI Readmission

eTable 2. Comparing the RSRRs from the Current CMS Model Without POA (Using Only the CoC Algorithm) to the CMS+POA Model Across Five Quintiles, HF Readmission

eTable 3. Comparing the RSRRs from the Current CMS Model Without POA (Using Only the CoC Algorithm) to the CMS+POA Model Across Five Quintiles, PN Readmission

eTable 4. Comparing the RSMRs from the Current CMS Model Without POA (Using Only the CoC Algorithm) to the CMS+POA Model Across Five Quintiles, AMI Mortality

eTable 5. Comparing the RSMRs from the Current CMS Model Without POA (Using Only the CoC Algorithm) to the CMS+POA Model Across Five Quintiles, HF Mortality

eTable 6. Comparing the RSMRs from the Current CMS Model Without POA (Using Only the CoC Algorithm) to the CMS+POA Model Across Five Quintiles, PN Mortality

eTable 7. Distributions of Differences in Risk-Standardized Rates With and Without POA for AMI, HF, and PN Readmission and Mortality Measures

This supplemental material has been provided by the authors to give readers additional information about their work.

© 2021 Triche EW et al. *JAMA Network Open.*

eAppendix 1. POA-Exempt Codes

Valid POA indicators are "Y" (Yes, Present on Admission), "N" (No, Not Present on Admission), "U" (Unknown), and "W" (Clinically undetermined). POA exempt codes are "categories and/or codes for circumstances regarding the healthcare encounter or factors influencing health status that do not represent a current disease or injury or are always present on admission"[1]. Review of the POA exempt list revealed additional instances, such as family history codes, in which POA status would not reflect a patient's health status at the time of admission.

To determine the list of POA codes that would be considered "always POA" and subsequently re-coded to POA = Y, we reviewed the POA-exempt codes list through a multi-step process with clinical input from a technical working group. First, we created groupings by code classification. Examples of groups include A00-B00 for "Certain infectious and parasitic diseases" and Q00-Q99 for "Congenital malformations, deformations, and chromosomal abnormalities." One group in particular, Z00-Z99 "Factors influencing health status and contact with health services," contained many clinically diverse codes, which we further delineated by commonality of content or coding purpose. All groupings were made in concert with input from a technical working group.

Next, we made initial recommendations for code groups to include as "always POA" and provided rationale for each recommendation based on coding guidance from the CMS ICD-10-CM Coding Guidelines.[1] The technical working group clinical experts provided clinical rationale for categorizing the POA-exempt codes as "always POA" or "don't count as POA" based on their expertise, the rationale from the ICD-10-CM Coding Guidelines, and our recommendations. We excluded POA-exempt codes from the "always POA" list based on any one of four criteria:

1) They were not relevant to the Medicare population 65 years or older, such as codes related to pregnancy, childbirth, and children's health statuses;

2) They could potentially be coded as a complication of care during an index admission or POA, such as exposure to toxic substances or medication overdoses;
3) They provided no relevant information about a patient's health status or reason for admission, such as family history codes or encounter codes, which indicate that a patient has an encounter for a procedure but does not specify that the procedure was performed; or
4) They were not mandatory for reporting. The latter exclusion pertained specifically to S00-T88 Injury, poisoning, and certain other consequences of external causes and V00-Y99 External Causes of Morbidity, which are claims collected for the purposes of injury research.

The majority of ICD-10 codes included in the POA-exempt list were subsequent, sequela, or congenital codes. Based on ICD-10-CM Coding Guidelines, we coded these types of codes as "always POA" because, by definition, subsequent and sequela codes should not be used for conditions acquired during a hospitalization in which the patient is receiving active treatment for that condition. Additional groups of codes were further reviewed by clinical experts to determine whether they should be counted as "always POA" or not.

Reference

1. 2018 ICD-10 CM and GEMs. 2018. Accessed March 10, 2020. https://www.cms.gov/Medicare/Coding/ICD10/2018-ICD-10-CM-and-GEMs

© 2021 Triche EW et al. *JAMA Network Open.*

eAppendix 2. Note on Quintile Shifts in eTables 1-6

eTables 1-6 report detailed quintile shifts in hospital performance by CAH status for the AMI, HF, and PN readmission and mortality measures. Overall, of the minority of hospitals that shifted quintiles, the vast majority only shifted one quintile. A small percentage shifted two quintiles. Compared to the three readmission measures, these shifts were more frequent among hospitals for the three mortality measures.

© 2021 Triche EW et al. *JAMA Network Open.*

eTable 1. Comparing the RSRRs from the current CMS model without POA (using only the CoC algorithm) to the CMS+POA model across five quintiles, AMI readmission

A. Comparing the RSRRs from the current CMS model without POA (using only the CoC algorithm) to the CMS+POA model across five quintiles for non-CAH ACHs (n=2,183), AMI readmission

**AMI Readmission (non-CAHs)**		**Current CMS Model Without POA – RSRR quintiles**				
		**1** (11.97, 14.92)	**2** (14.92, 15.49)	**3** (15.49, 15.97)	**4** (15.97, 16.59)	**5** (16.59, 21.90)
**CMS Model + POA – RSRR quintiles**	**1** (12.02, 14.95)	415	21	0	0	0
	**2** (14.95, 15.49)	21	388	28	0	0
	**3** (15.49, 15.97)	0	28	377	32	0
	**4** (15.97, 16.56)	0	0	32	381	24
	**5** (16.56, 21.96)	0	0	0	24	412

Note: For non-CAH ACH in AMI readmission measure, 991 hospitals with fewer than 25 claims, were excluded from the above table analysis.

**Ranges represent the upper and lower limits of each quintile.*

B. Comparing the RSRRs from the current CMS model without POA (using only the CoC algorithm) to the CMS+POA model across five quintiles for CAHs (n=14), AMI readmission

**AMI Readmission (CAHs)**		**Current CMS Model Without POA – RSRR quintiles**				
		**1** (14.89,14.91)	**2** (15.07,15.3)	**3** (15.31,15.74)	**4** (15.77,16.12)	**5** (16.22,16.55)
**CMS Model + POA - RSRR quintiles**	**1** (14.91,14.92)	2	0	0	0	0
	**2** (15.07,15.38)	0	2	1	0	0
	**3** (15.45,15.72)	0	1	2	0	0
	**4** (15.75,16.21)	0	0	0	2	1
	**5** (16.28,16.51)	0	0	0	1	2

Note: For CAH in AMI readmission measure, 962 hospitals with fewer than 25 claims were excluded from the above table analysis.

© 2021 Triche EW et al. *JAMA Network Open.*

eTable 2. Comparing the RSRRs from the current CMS model without POA (using only the CoC algorithm) to the CMS+POA model across five quintiles, HF readmission

A. Comparing the RSRRs from the current CMS model without POA (using only the CoC algorithm) to the CMS+POA model across five quintiles for non-CAH ACHs (n=3,076), HF readmission

**HF Readmission (non-CAHs)**		**Current CMS Model Without POA – RSRR quintiles**				
		**1** (15.63, 20.37)	**2** (20.37, 21.25)	**3** (21.25, 22.02)	**4** (22.02, 23.04)	**5** (23.04, 30.08)
**CMS Model + POA – RSRR quintiles**	**1** (15.39, 20.24)	599	16	0	0	0
	**2** (20.24, 21.20)	16	567	32	0	0
	**3** (21.21, 22.01)	0	32	544	40	0
	**4** (21.01, 23.12)	0	0	40	550	25
	**5** (23.12, 30.94)	0	0	0	25	590

Note: For non-CAH ACHs in HF readmission measure, 251 hospitals with fewer than 25 claims, were excluded from the above table analysis.

B. Comparing the RSRRs from the current CMS model without POA (using only the CoC algorithm) to the CMS+POA model across five quintiles for CAHs (n=694), HF readmission

**HF Readmission (CAHs)**		**Current CMS Model Without POA – RSRR quintiles**				
		**1** (18.54, 20.74)	**2** (20.75, 21.27)	**3** (21.28, 21.77)	**4** (21.77, 22.40)	**5** (22.40, 25.95)
**CMS Model + POA – RSRR quintiles**	**1** (18.46, 20.74)	133	5	0	0	0
	**2** (20.75, 21.33)	5	127	7	0	0
	**3** (21.34, 21.86)	0	7	123	9	0
	**4** (21.87, 22.55)	0	0	9	125	5
	**5** (22.55, 26.56)	0	0	0	5	134

Note: For CAHs in HF readmission measure, 644 hospitals with fewer than 25 claims, were excluded from the above table analysis.

© 2021 Triche EW et al. *JAMA Network Open.*

eTable 3. Comparing the RSRRs from the current CMS Model Without POA (Using Only the CoC Algorithm) to the CMS+POA Model Across Five Quintiles, PN Readmission

A. Comparing the RSRRs from the current CMS model without POA (using only the CoC algorithm) to the CMS+POA model across five quintiles for non-CAH ACHs (n=3,166), PN readmission

PN Readmission (Non-CAHs)		Current CMS Model Without POA – RSRR quintiles				
		1 (12.66, 15.64)	2 (15.64, 16.33)	3 (16.33, 16.97)	4 (16.97, 17.79)	5 (17.79, 23.81)
CMS Model + POA – RSRR quintiles	1 (12.63, 15.60)	604	29	0	0	0
	2 (15.60, 16.31)	29	565	39	0	0
	3 (16.31, 16.94)	0	39	547	48	0
	4 (16.94, 17.76)	0	0	48	549	36
	5 (17.77, 24.60)	0	0	0	36	597

Note: For ACH in PN readmission measure, 207 hospitals with fewer than 25 claims, had been excluded from the above table analysis.

B. Comparing the RSRRs from the current CMS model without POA (using only the CoC algorithm) to the CMS+POA model across five quintiles for CAHs (n=1,088), PN readmission

PN Readmission (CAHs)		Current CMS Model Without POA – RSRR quintiles				
		1 (14.14, 15.75)	2 (15.75, 16.15)	3 (16.15, 16.47)	4 (16.48, 16.92)	5 (16.94, 19.31)
CMS Model + POA – RSRR quintiles	1 (14.17, 15.81)	207	10	0	0	0
	2 (15.81, 16.22)	10	184	24	0	0
	3 (16.22, 16.58)	0	24	176	18	0
	4 (16.58, 17.05)	0	0	18	189	11
	5 (17.05, 19.64)	0	0	0	11	206

Note: For CAH in PN readmission measure, 266 hospitals with fewer than 25 claims, had been excluded from the above table analysis.

© 2021 Triche EW et al. *JAMA Network Open.*

eTable 4. Comparing the RSMRs from the current CMS model without POA (using only the CoC algorithm) to the CMS+POA model across five quintiles, AMI mortality

A. Comparing the RSMRs from the current CMS model without POA (using only the CoC algorithm) to the CMS+POA model across five quintiles for non-CAH ACHs (n=2,352), AMI mortality

AMI Mortality (Non-CAHs)		Current CMS Model Without POA – RSMR quintiles				
		**1** (8.83, 11.89)	**2** (11.89, 12.50)	**3** (12.50, 13.02)	**4** (13.02, 13.71)	**5** (13.71, 17.16)
**CMS Model + POA – RSMR quintiles**	**1** (9.12, 11.87)	409	60	1	0	0
	**2** (11.87, 12.49)	60	320	86	5	0
	**3** (12.49, 12.98)	1	88	288	92	1
	**4** (12.98, 13.71)	0	3	93	303	72
	**5** (13.71, 18.08)	0	0	2	71	397

Note: For ACH in AMI mortality measure, 868 hospitals with fewer than 25 claims, were excluded from the above table analysis.

B. Comparing the RSMRs from the current CMS model without POA (using only the CoC algorithm) to the CMS+POA model across five quintiles for CAHs (n=35), AMI mortality

AMI Mortality (CAHs)		Current CMS Model Without POA – RSMR quintiles				
		**1** (11.88, 12.65)	**2** (12.65, 12.87)	**3** (12.92, 13.13)	**4** (13.21, 13.51)	**5** (13.55, 14.66)
**CMS Model + POA – RSMR quintiles**	**1** (11.82, 12.74)	2	4	1	0	0
	**2** (12.79, 13.15)	4	2	1	0	0
	**3** (13.24, 13.29)	1	0	2	4	0
	**4** (13.30, 13.82)	0	1	3	3	0
	**5** (13.82, 15.06)	0	0	0	0	7

Note: For CAH in AMI mortality measure, 1,049 hospitals with fewer than 25 claims, were excluded from the above table analysis.

© 2021 Triche EW et al. *JAMA Network Open.*

eTable 5. Comparing the RSMRs from the current CMS Model Without POA (Using Only the CoC Algorithm) to the CMS+POA Model Across Five Quintiles, HF Mortality

A. Comparing the RSMRs from the current CMS model without POA (using only the CoC algorithm) to the CMS+POA model across five quintiles for non-CAH ACHs (n=3,056), HF mortality

HF Mortality (Non-CAHs)		Current CMS Model Without POA – RSMR quintiles				
		1 (5.45, 10.00)	2 (10.00, 10.98)	3 (10.98, 11.79)	4 (11.80, 12.84)	5 (12.84, 18.78)
CMS Model + POA – RSMR quintiles	1 (5.44, 9.94)	570	41	0	0	0
	2 (9.94, 10.90)	41	498	72	0	0
	3 (10.91, 11.75)	0	72	453	87	0
	4 (11.76, 12.79)	0	0	87	468	56
	5 (12.79, 19.86)	0	0	0	56	555

Note: For non-CAH ACH in HF mortality measure, 269 hospitals with fewer than 25 claims, were excluded from the above table analysis.

Table B5b. Comparing the RSMRs from the current CMS model without POA (using only the CoC algorithm) to the CMS+POA model across five quintiles for CAHs (n=634), HF mortality

HF Mortality (CAHs)		Current CMS Model Without POA – RSMR quintiles				
		1 (9.01, 10.91)	2 (10.91, 11.69)	3 (11.70, 12.38)	4 (12.38, 13.23)	5 (13.24, 16.98)
CMS Model + POA – RSMR quintiles	1 (9.11, 11.05)	117	9	0	0	0
	2 (11.05, 11.83)	9	106	12	0	0
	3 (11.83, 12.53)	0	12	99	16	0
	4 (12.53, 13.54)	0	0	16	98	13
	5 (13.54, 17.32)	0	0	0	13	114

Note: For CAH in HF mortality measure, 702 hospitals with fewer than 25 claims, were excluded from the above table analysis.

© 2021 Triche EW et al. *JAMA Network Open.*

eTable 6. Comparing the RSMRs from the Current CMS Model Without POA (Using Only the CoC Algorithm) to the CMS+POA Model Across Five Quintiles, PN Mortality

A. Comparing the RSMRs from the current CMS model without POA (using only the CoC algorithm) to the CMS+POA model across five quintiles for non-CAH ACHs (n=3,156), PN mortality

PN Mortality (Non-CAHs)		Current CMS Model Without POA – RSMR quintiles				
		1 (8.06, 13.81)	2 (13.81, 15.04)	3 (15.04, 16.06)	4 (16.06, 17.47)	5 (17.47, 25.79)
CMS Model + POA – RSMR quintiles	1 (7.31, 13.42)	547	84	2	0	0
	2 (13.42, 14.70)	84	421	124	4	0
	3 (14.70, 15.87)	2	123	362	141	5
	4 (15.87, 17.37)	0	5	139	388	101
	5 (17.37, 29.60)	0	0	6	100	527

Note: For non-CAH ACHs in PN mortality measure, 205 hospitals with fewer than 25 claims, were excluded from the above table analysis.

Table B6b. Comparing the RSMRs from the current CMS model without POA (using only the CoC algorithm) to the CMS+POA model across five quintiles for CAHs (n=1,089), PN mortality

PN Mortality (CAHs)		Current CMS Model Without POA – RSMR quintiles				
		1 (10.63, 14.37)	2 (14.37, 15.17)	3 (15.17, 15.99)	4 (15.99, 17.14)	5 (17.14, 25.04)
CMS Model + POA – RSMR quintiles	1 (11.35, 14.69)	193	24	0	0	0
	2 (14.70, 15.71)	23	152	43	0	0
	3 (15.71, 16.79)	1	39	129	48	1
	4 (16.81, 18.27)	0	3	44	134	37
	5 (18.29, 27.44)	0	0	2	36	180

Note: For CAH in PN mortality measure, 264 hospitals with fewer than 25 claims, were excluded from the above table analysis.

© 2021 Triche EW et al. *JAMA Network Open.*

eTable 7. Distributions of Differences in Risk-Standardized Rates with and without POA for AMI, HF, and PN Readmission and Mortality Measures

Measure	Hospitals with ≥25 Claims (N)	Mean Risk-Standardized Rate Difference (%) (SD)	Median Risk-Standardized Rate Difference (%)	25th Percentile Risk-Standardized Rate Difference (%)	75th Percentile Risk-Standardized Rate Difference (%)
AMI Readmission	2,197	<0.001 (0.11)	-0.007	-0.066	0.057
HF Readmission	3,770	0.002 (0.16)	-0.008	-0.101	0.095
PN Readmission	4,254	<0.001 (0.12)	0.008	-0.069	0.069
AMI Mortality	2,387	-0.013 (0.32)	-0.022	-0.212	0.179
HF Mortality	3,690	-0.007 (0.28)	-0.001	-0.176	0.168
PN Mortality	4,254	0.042 (0.77)	-0.028	-0.471	0.468

Table C1 reports the hospital-level results among hospitals with 25 or more admissions. Mean differences in hospital RSRRs between models with and without POA were very small for all three readmission measures, with the largest mean difference of 0.002% (SD=0.16) for HF readmission. The lower and upper quartiles of differences in RSRRs ranged from -0.066 to 0.057% for AMI, from -0.101 to 0.095% for HF, and from -0.069 to 0.069% for PN.

For the AMI, HF, and PN mortality measures, the differences in RSMRs between models with and without POA indicators were relatively larger and had wider ranges than the differences in RSRRs. The mean differences in RSMRs were negative for AMI (-0.013%) and HF (-0.007%). Specifically, the lower and upper quartiles of differences in RSMRs ranged from -0.212 to 0.179% for AMI, from –0.176 to 0.168% for HF, and from -0.471 to 0.468% for PN.

© 2021 Triche EW et al. *JAMA Network Open.*
